# A Sheep-Shearing Trust; John B. Murphy, M. D., LL. D., President

**Published:** 1903-06

**Authors:** 


### TEXAS MEDICAL JOURNAL.

AUSTIN, TEXAS.

A MONTHLY JOURNAL OF MEDICINE AND SURGERY.

EDITED AND PUBLISHED BY
F. E. DANIEL, M. D.

ASSOCIATE EDITORS:

WITTEN BOOTH RUSS, M. D., San Antonio, Texas.

P. M. PAYNE, M. D., Brownwood, Texas.

Published Monthly at Austin, Texas. Subscription price $1.00 a year in advance.

Eastern Representative: John Guy Monihan, St. Paul Building, 220 Broadway, New York City.

Official organ of the the West Texas Medical Association, the Houston District Medical Association, the Austin District Medical Society, the Brazos Valley Medical Association, the Galveston County Medical Society, and several others.

### A SHEEP-SHEARING TRUST; JOHN B. MURPHY, M. D., LL. D., PRESIDENT.

"Once I was as pure as the snow, but I fell;
Fell, like the snowflakes, from heaven to hell."

Said the white man to the negro: "Get your hook and line and pole and some bait. You may go with me fishing, and I will give you half you catch."

**Commercialism in Medicine Gone Mad.**

"It's English, you know." (Why English?) The "Christian Hospital, Chicago, incorporated under the laws of Illinois," has been organized, with the great Jno. B. Murphy, M. D., LL. D., as president. They issue an "English Hospital certificate" (in Latin), to all who pay them $25 for it and appoint them a member of the hospital "staff." The scheme seems to be a systematic and very extensive drumming up of patients to be sent to the Christian Hospital, where it is proposed to fleece them and divide the fleece with the sender. Any member of the "staff" can have the privilege (on payment of $25) of sending patients to the great Murphy and his associates, to be operated on on shares. As to the "associates,"

they are so completely overshadowed by the great Murphy as to be not worth mentioning—especially as they are unknown to fame, at least I never heard of any of them before I received one of the decoy diplomas (made out to Dr. A. C. Bernays). I note that in the list of names following that of the great Murphy—like a tail to his kite, are three "Woods," a "Schwarz," a "Baker," and some other Woods, if he could; not worth while, really. From the number of these "English certificates," nearly all of which are made out in the name of some famous surgeon, such as Francis Delafield, Jno. B. Deavers, A. C. Bernays—sent me by Texas subscribers to the "Red Back," with the request to "go for Murphy—" I assume that every doctor in Texas, and perhaps everywhere else, received an invitation to "walk into my parlor"—and get skinned. One esteemed friend of the "Red Back" sent me *his* invitation, with the following endorsements:

"Dear Doctor Daniel: I send you herewith some matter which is self-explanatory. This is, doubtless, the opportunity of my life, if I would bite at it, but being of a generous and unselfish disposition I pass it on to you, in the belief that such wonderful opportunities are purposely withheld from medical editors. You will note that this is mailed in the same envelope in which it is received, I not having anything *large* enough to hold so important an offer as this. I am sure you will appreciate the great sacrifice I make in your behalf.

Sincerely yours,

A. J. S———."

This "diploma," or "English Hospital certificate" (Latin), was made out in the name of Dr. Bernays. I forwarded it to Dr. Bernays and asked if it were done by his authority. Dr. Bernays wrote as follows:

"St. Louis, Mo., May 19, 1903.

Dear Doctor Daniel: About two weeks or ten days ago I was appointed 'honorary' member of the staff by the Christian Hospital, and seeing J. B. Murphy's picture and signature as chief or president of staff, I accepted the 'honor' thrust on me. It looks now like a 'fake,' and the use of my name as they are using it is without my knowledge or consent. I am opposed to all manner of commercialism in our profession. I feel much hurt and have written to Murphy asking how he is connected with this thing, and I am sure that I would have refused to be 'honored'? had it not been for Murphy's connection with the staff as its president.

Yours truly,

A. C. Bernays."

Another one of the decoys was sent me, made out to Prof. Francis Delafield. I forwarded it to him, with request to know if he had authorized it. Dr. Delafield wrote me as follows:

"12 West Thirty-second Street,
NEW YORK, May 26, 1903.

*Dr. F. E. Daniel, Editor Texas Medical Journal.*

DEAR SIR: Some time ago I received a letter from the Christian Hospital, telling me they had appointed me as a member of their staff. I at once wrote to them, declining the appointment.

Since then I have received letters like yours. The whole scheme seems to be a curious one, and I should be glad to have it made public that I have nothing to do with it.

Yours truly,

FRANCIS DELAFIELD."

I'm going to give these fellows the benefit of a free advertisement. I reproduce the letter sent out with the decoy diploma, and I submit it without comment, further than to say: "Well—I'll be doggoned." It paralyzes my speech centers. Notice the inducements offered: An "English Hospital certificate," showing you (any doctor in Texas or elsewhere, who will bite) to be a member of its "staff," will be "a stronger drawing card than an ordinary diploma, as it indicates a *higher* attainment." The pocket size certificate ($5.00), if *"judiciously displayed,"* will bring more dollars than—well, here it is:

"CHICAGO, ILL., May 12, 1903.

"DEAR DOCTOR: Enclosed we hand you announcement of our hospital, together with one of our physician's application blanks for you to fill out and return to us, should you wish to be appointed a member of our staff.

The advantages and benefits to be obtained by joining us are briefly stated as follows, viz.:

1. Our certificate of membership (see fac-simile) is artistically designed, and executed in the highest style of the lithographers' art. Neatly framed and hung on the walls of your reception room, it *imparts confidence* to all visitors and patients, and is a much *stronger* drawing card than an ordinary diploma, as it indicates a *higher* attainment.

2. We furnish free with each of our certificates (or at $5.00 when ordered separately) a solid gold lapel button of special *Red Cross* design in enamel, and the words "Christian Hospital, Chicago" in circular form around the red cross; also a neat pocket membership ticket. This alone, if displayed judiciously, will bring more dollars annually than the cost of your membership.

*"Dollars to Doctors,"* Dr. N. E. Wood's new book on "Case-Taking and Fee-Getting," will also be given free to all members who take our $20 or $25 certificates.

3. We will agree to pay you a cash commission of 50 per cent. of all surgical fees, and 25 per cent. of all medical fees received from patients you *bring* or *send* to us. This, of course, *will be held strictly private and confidential.* Your professional interests as well as our own demand this.

4. We have a large number of the best Chicago surgeons and specialists in all branches on our staff, therefore *you are assured* the surgical and hospital cases you send us will receive the most expert treatment, equal to the best to be obtained in any medical center. You can bring in a case and spend a few days with us and take a *free post-graduate course,* and the patient (through us) will pay all your expenses.

5. We have a *Lying-in Department* in connection, and invite you to refer maternity cases, "unfortunate" or otherwise, who are seeking the quiet seclusion of such a necessary institution.

6. We usually appoint but one physician in each locality, so if you desire this appointment it will be necessary to get your application in early, for if not accepted within a reasonable time, it will be offered to one of your neighbors.

Yours fraternally,

CHRISTIAN HOSPITAL,
N. News Wood, A. M., M. D.,
President and Superintendent."

Don't forget that you get a button (not a Murphy's button, but a red-cross button), which must also be "judiciously displayed."

This is about the boldest piece of effrontry I ever saw. These fellows are evidently using Murphy's name as a bait, and they count confidently and correctly on the vanity and weakness of many in the ranks of the profession who will bite at it. It is very much like the white man's proposition to the negro. The victims (sheep), are to be sent to these wolves, to be sheared to the Queen's taste, and the sender can "spend a few days in Chicago and take a post-graduate course" at their expense.

And Murphy, an unusually gifted young surgeon, who has startled the world by the brilliancy of his genius and his operations; a young man whose fame and fortune were secured by virtue of his anastromosing device in resections of the intestines; who was the center of interest at the Tenth International Medical Congress in St. Petersburg, while he read a paper giving an account of his successful ligation of the subclavian artery for gun-shot wound, a courtesy unprecedented; an exemplar to the younger physicians, now stoops from his exalted position to engage in what I conceive to be a shameful scheme to hoodwink the country doctor and skin the confiding and unsuspicious afflicted; a species of quackery unsurpassed by the barefaced Niles Hospital scheme. Shame! Shame!

